# Localized Increased Permeability of Blood–Brain Barrier for Antibody Conjugates in the Cuprizone Model of Demyelination

**DOI:** 10.3390/ijms241612688

**Published:** 2023-08-11

**Authors:** Tatiana Abakumova, Anastasia Kuzkina, Philipp Koshkin, Daria Pozdeeva, Maxim Abakumov, Pavel Melnikov, Klavdia Ionova, Ilia Gubskii, Olga Gurina, Natalia Nukolova, Vladimir Chekhonin

**Affiliations:** 1 Department of Synthetic Neurotechnology, Pirogov Russian National Research Medical University, Moscow 117997, Russia; 2 Faculty of Medicine, Sechenov First Medical University, Moscow 119991, Russia; 3 Department of Immunochemistry, V. Serbsky National Medical Research Center for Psychiatry and Narcology, Moscow 119034, Russia; 4 Department of Medicine and Biology, Chair of Medical Nanotechnology, Pirogov Russian National Research Medical University, Moscow 117997, Russia; 5 Laboratory of Biomedical Nanomaterials, National University of Science and Technology MISIS, Moscow 119049, Russia; 6 Massachusetts Institute of Technology, Koch Institute for Integrative Cancer Research, Cambridge, MA 02139, USA

**Keywords:** cuprizone, BBB permeability, demyelination, antibody conjugates, Gd–DTPA, MRI, Evans blue

## Abstract

The development of new neurotherapeutics depends on appropriate animal models being chosen in preclinical studies. The cuprizone model is an effective tool for studying demyelination and remyelination processes in the brain, but blood–brain barrier (BBB) integrity in the cuprizone model is still a topic for debate. Several publications claim that the BBB remains intact during cuprizone-induced demyelination; others demonstrate results that could explain the increased BBB permeability. In this study, we aim to analyze the permeability of the BBB for different macromolecules, particularly antibody conjugates, in a cuprizone-induced model of demyelination. We compared the traditional approach using Evans blue injection with subsequent dye extraction and detection of antibody conjugates using magnetic resonance imaging (MRI) and confocal microscopy to analyze BBB permeability in the cuprizone model. First, we validated our model of demyelination by performing T2-weighted MRI, diffusion tensor imaging, quantitative rt-PCR to detect changes in mRNA expression of myelin basic protein and proteolipid protein, and Luxol fast blue histological staining of myelin. Intraperitoneal injection of Evans blue did not result in any differences between the fluorescent signal in the brain of healthy and cuprizone-treated mice (IVIS analysis with subsequent dye extraction). In contrast, intravenous injection of antibody conjugates (anti-GFAP or non-specific IgG) after 4 weeks of a cuprizone diet demonstrated accumulation in the corpus callosum of cuprizone-treated mice both by contrast-enhanced MRI (for gadolinium-labeled antibodies) and by fluorescence microscopy (for Alexa488-labeled antibodies). Our results suggest that the methods with better sensitivity could detect the accumulation of macromolecules (such as fluorescent-labeled or gadolinium-labeled antibody conjugates) in the brain, suggesting a local BBB disruption in the demyelinating area. These findings support previous investigations that questioned BBB integrity in the cuprizone model and demonstrate the possibility of delivering antibody conjugates to the corpus callosum of cuprizone-treated mice.

## 1. Introduction

Several devastating central nervous system (CNS) diseases are associated with demyelination and remyelination processes. The most frequent demyelinating disease is multiple sclerosis (MS), characterized by recurrent episodes of demyelination resulting in neuro-axonal degeneration. Various models have been developed to understand the underlying mechanisms of these processes [1]. Among toxin-induced models of demyelination, the cuprizone-induced demyelination model attracts prominent interest due to relatively good reproducibility in contrast to other models of MS [2]. The cuprizone diet causes primary oligodendrocyte apoptosis and secondary demyelination of nerve fibers. The demyelination is accompanied by mitochondrial dysfunction and oligodendrocyte loss and results in the formation of multiple lesions in different brain regions enriched by white (corpus callosum, superior cerebellar peduncles) and grey matter (cortex, cerebrum, and cerebellum). Demyelination and inflammation processes in the CNS are accompanied by reactive astrogliosis, peripheral macrophage recruitment, and progenitor cell activation [3,4,5]. Thus, the cuprizone model of demyelination triggers all of these complex processes in the CNS. 

Historically, a majority of publications suggested that the blood–brain barrier (BBB) stays intact during cuprizone intoxication [6]. However, this statement is based on a few studies conducted in the 1980s and earlier [7,8,9]. Namely, in 1969, Suzuki and colleagues injected toluidine dye Trypan blue into two cuprizone-intoxicated mice with encephalopathy symptoms to check the BBB integrity in the treated mice [8]. They did not detect any accumulation of Trypan blue in the mice’s brains and concluded that the BBB was not compromised. But the following peculiarities of this investigation should be taken into consideration: (1) the use of 3–4-week-old Swiss Webster mice (major publications used C57Bl6 mice in their experiments), (2) the cuprizone treatment of mice lasted for only 2 weeks, and (3) insufficient number of animals in the study (only two mice out of forty demonstrated encephalopathy symptoms during the cuprizone diet and were used for subsequent investigation of BBB integrity). However, it was later shown that the severity and reproducibility of demyelination strongly depend on animal strain and age. Hiremath et al. (1998) published a key study that determined that cuprizone feeding of 8-week-old C57BL/6 mice consistently induced demyelination with minimal clinical toxicity [10]. Since then, the cuprizone-induced demyelination model using C57BL/6 mice has become the most used variant of the cuprizone model due to its relatively high reproducibility. Moreover, the duration of diet exposure plays a crucial role in demyelination; subsequent studies have shown that maximum demyelination was achieved no earlier than 4–6 weeks on a cuprizone diet. Later, the permeability of the BBB for macromolecules in the murine cuprizone model was studied by two groups: Akira Kondo; and Suzuki, Bakker, and Ludwin [7,9]. They showed that the BBB in the area of demyelinated nerve fibers is not permeable to horseradish peroxidase (HRP, 40 kDa) within 30 or 60 min after intravenous injection of HRP. Immunochemical analysis of brain slices using antiserum to detect serum proteins reaffirmed that there was no BBB breakdown [9]. The result was supported by a panel of different methods (e.g., electronic microscopy, histological evaluation). In these studies, the authors used a brain injury model as a positive control for increased BBB permeability.

Later on, several publications demonstrated results that could be explained by the increased permeability of the BBB in the cuprizone model. For example, Hedayatpour et al. showed that intravenously transplanted adipose mesenchymal stem cells could migrate into demyelinated lesions in the murine cuprizone model [11]. However, it is known that intravenously administrated cells are unlikely to migrate across the healthy BBB because the BBB prevents access of cells and a majority of molecules to the parenchyma. The authors suggested that focal demyelination might induce chemoattractant signals that promote the accumulation of mesenchymal stem cells in the brain [11]. But it could also be explained by the disruption of the BBB in the demyelinated area in cuprizone-treated mice. Also, peripheral macrophages and a low number of recruited T cells were observed in the corpus callosum of cuprizone-treated mice [3,5]. Thus, these findings could indicate increased BBB permeability in the cuprizone model. 

Currently, BBB integrity in the cuprizone model remains a topic of debate. Some researchers claim that the BBB remains intact in the cuprizone model, while others have observed signs of increased BBB permeability in their studies. Monokesh K. Sen et al. [12] state that the BBB remains intact based on other researchers’ recent histological and proteomic investigations [13,14,15]. Tejedor et al. did not observe any accumulation of Evans blue dye in cuprizone-treated mice [15], while Shelestak et al. observed accumulation of Evans blue in an early phase of the cuprizone model and suggested a mechanism of increased BBB permeability [16]. Berghoff et al. also questioned BBB integrity and proposed that BBB hyperpermeability precedes demyelination in the cuprizone model [17]. Martin Zirngibl et al. (2022) asserted that the BBB remains largely intact in the cuprizone model but also suggested that altered BBB integrity may permit infiltration of leukocytes [18]. These contradictory results led us to the idea that the increased permeability of the BBB for macromolecules in the cuprizone model might be subtle and localized and could be detectable using sensitive methods, such as molecular magnetic resonance imaging (MRI) or confocal microscopy. Moreover, tracers with high fluorescence intensity can improve the chances of detecting the locally increased permeability in the BBB during cuprizone-induced demyelination. Also, injection of targeted molecules (such as anti-GFAP conjugates) can improve detection since non-specific conjugates (non-specific IgG) demonstrate minor accumulation in disrupted BBBs in other models [19,20,21] and could provide false-negative results.

Therefore, in this study, we investigated BBB permeability for macromolecules using injections of Evans blue, Alexa Fluor™ 488-labeled specific antibodies to GFAP, and gadolinium-labeled antibody conjugates in healthy and cuprizone-treated mice. We used an optimized protocol of the cuprizone model and evaluated the accumulation of these tracers by immunohistochemical and MRI analysis in the brains of animals. Our results suggest that BBB integrity is compromised in the white matter of the brains of cuprizone-treated mice in the cuprizone model of demyelination (in particular, in the corpus callosum).

## 2. Results

### 2.1. Validation of the Cuprizone Model of Demyelination in Mice

**Figure 1:**
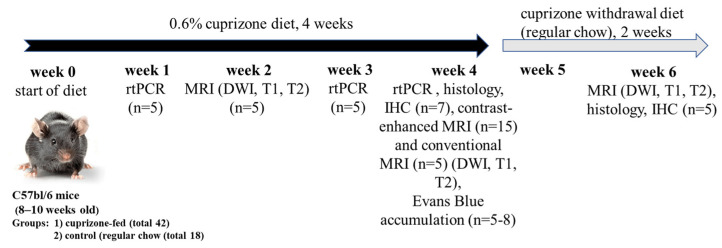
Experimental design of cuprizone model of demyelination and further remyelination (after cuprizone withdrawal) and its validation and characterization (n = number of mice in experimental group).

*Experimental design*: Demyelination of nerve fibers in the brains of male C57BL/6 mice was induced by cuprizone intoxication using a rodent chow containing 0.6% cuprizone [22]. Previously, we compared different doses of cuprizone to validate the model. However, the 0.2% cuprizone model did not result in any notable pathological changes, so the dose was increased to 0.6% to observe the demyelination of nerve fibers comparable to other published results [22]. The experimental design and number of animals per group are indicated in Figure 1. The cuprizone-treated animals demonstrated a slight decrease in body weight, and by the 4th week of the cuprizone diet, their weight loss differed compared to healthy mice (24.3 ± 0.8 g vs. 27.8 ± 0.3 g).

**Figure 2:**
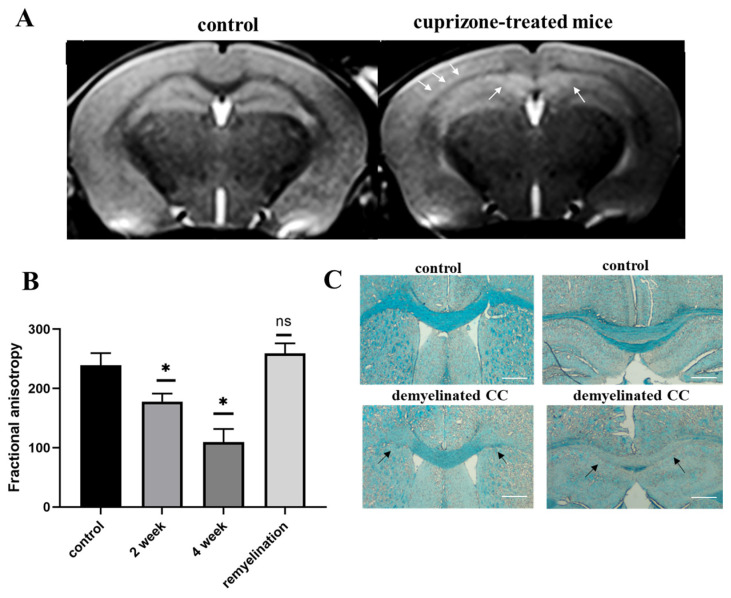
(**A**) T2-weighted MRI images of the corpus callosum (CC) of healthy and cuprizone-treated mice with demyelinated CC after 4 weeks of cuprizone diet (pathological changes are marked with arrows). (**B**) Fractional anisotropy of the corpus callosum of mice during the cuprizone diet (after 2 and 4 weeks of cuprizone diet) and after its termination (2 weeks after cuprizone withdrawal); number of animals per group is 5. (**C**) Representative images of the brain cross sections of healthy and cuprizone-treated mice by Luxol Fast Blue staining of myelin in the corpus callosum (demyelinated regions are marked with arrows). Scale bar: 500 µm. * *p*-value < 0.05, ns—non-significant.

*MRI and histological evaluation of cuprizone-induced demyelination:* T2-weighted MRI images revealed pathological lesions in the corpus callosum of mice treated with cuprizone compared to non-treated mice during the 4th week of the diet (Figure 2A). As expected, 2 weeks after the termination of the cuprizone diet, the signal intensity of T2-weighted MRI images increased, reflecting the remyelination process. We also performed a diffusion MRI to determine brain fiber structure using water diffusion properties as a probe (Figure S1). The calculated values of the fractional anisotropy obtained from diffusion tensor MRI imaging reflect the disturbance of the cellular structure and are widely used to measure connectivity in the brain and estimate white matter damage (i.e., demyelination). In our study, fractional anisotropy in the brains of cuprizone-treated animals showed a two-fold decrease in the 4th week of the cuprizone diet, followed by restoration to its baseline values during remyelination (Figure 2B). Histological staining of myelin using Luxol fast blue also demonstrated demyelination in the brains of cuprizone-treated mice. Figure 2C shows that the amount of myelin in the corpus callosum was significantly reduced in the treated animals after 4 weeks of the cuprizone diet (Figure 2C and Figure S7).

**Figure 3:**
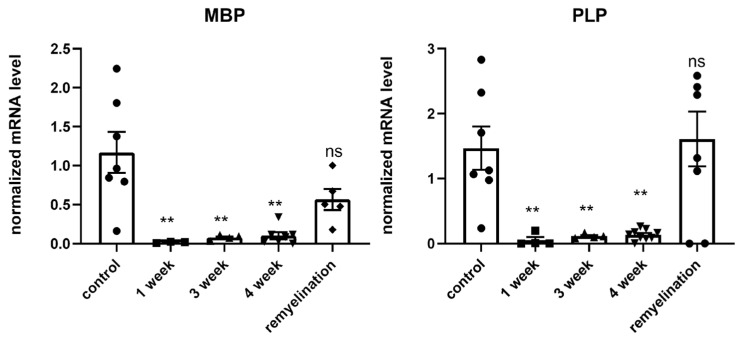
mRNA expression of MBP and PLP in brain hemispheres during the cuprizone diet (1, 3, 4 week of diet) and after its termination (2 weeks after cuprizone withdrawal) as detected by rt-PCR. Data are presented as mean ± SEM. ** *p*-value < 0.01, ns—non-significant.

*Analysis of mRNA levels of myelin proteins by real-time polymerase chain reaction (rt-PCR):* Gene expression of myelin basic proteins (MBP) and proteolipid proteins (PLP), which together account for 90% of all myelin proteins in the brain, were analyzed by rt-PCR. Figure 3 demonstrates the significant reduction in MBP and PLP expression levels after the first week of the cuprizone diet. Throughout the entire cuprizone-feeding period (4 weeks), there was a decrease in the expression levels of these proteins, which reflects demyelination. The expression levels increased or returned to their initial levels after the termination of the diet. These results also corroborate that the cuprizone diet used in this study reflects the demyelination process in the CNS, as observed by others [23,24].

*Immunostaining of GFAP and VEGFR2 of the cuprizone-injured brain:* It is known that demyelination is accompanied by increased astrocyte reactivity; inflammation and demyelination processes in glia are characterized by elevated GFAP expression (Figure S4) [4,25,26,27]. Using immunofluorescence staining with pAb anti-GFAP, we detected increased GFAP expression after 4 weeks of cuprizone exposure, indicating a robust glial reaction (Figure S4). However, the astroglial reactivity did not normalize after cuprizone withdrawal, and the remyelination was also accompanied by increased GFAP expression (Figure S4C). The high level of GFAP expression during demyelination was taken into account in our further experiments, where we analyzed the vascular permeability of the BBB during demyelination using specific monoclonal antibodies (mAb) to GFAP.

**Figure 4:**
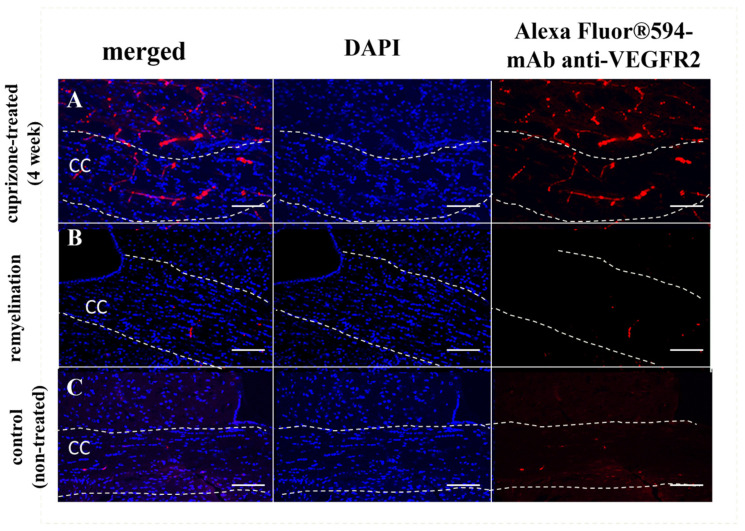
Immunofluorescent staining of the corpus callosum (CC) cross sections with mAb anti-VEGFR2 (red) of the cuprizone-treated mice during the 4th week of the diet ((**A**) demyelination) and after its termination ((**B**) remyelination) in comparison with control ((**C**) healthy mice). Nuclei are counterstained with DAPI (blue). Scale bar is 100 µm.

*Expression of the VEGF receptor (VEGFR2)*: We also investigated the expression of the VEGF receptor (VEGFR2) during demyelination and remyelination using immunofluorescent staining. It is known that VEGF and its receptors are the main regulators of angiogenesis and vascular permeability. It has been shown that high expression of one of the VEGF receptors, Flt-1 (VEGFR2), in endothelial cells and astrocytes is associated with micronecrosis during a brain injury and plays a role in the disruption of the BBB [28]. In our study, we detected high levels of VEGFR2 expression mostly in the vessels of brain cross sections of cuprizone-treated mice during the 4th week of the diet. This elevated VEGFR2 expression during the demyelination stage probably reflects the cuprizone-mediated damage and apoptosis of oligodendrocytes in the corpus callosum and might be associated with BBB disruption (Figure 4A). After cuprizone withdrawal, the expression of VEGFR2 in the brain reduced, reflecting the recovery process (Figure 4B) [29].

### 2.2. Detection of Evans Blue Dye in the Brain of Cuprizone-Treated Mice

To compare our results with previous studies on BBB permeability, we followed the conventional protocol using Evans blue dye [30]. Evans blue is an isomer of Trypan blue with a higher half-life time in the blood (>120 min) and, similar to Trypan blue, it binds to plasma proteins and reflects albumin leakage through the impaired BBB [31]. After i.v. injection of Evans blue, first, whole brain samples were scanned using an IVIS imaging system (Figure S3A). Then, the dye was extracted from the brain homogenates, and its concentration was analyzed by a VictorX3 fluorescence reader (PerkinElmer, Waltham, MA, USA). Both methods detected no fluorescence in the control (intact brain) and experimental (cuprizone-treated) groups of mice. We assumed that the sensitivity of the IVIS Spectrum CT or VictorX3 fluorescence reader was insufficient for detecting small amounts of albumin-bound Evans blue that might leak through the disturbed BBB. In comparison, fluorescence analysis of the brain with significant BBB impairment (glioma C6) demonstrated a significant fluorescent signal as detected by IVIS Spectrum CT (Supplementary Material, Figure S3B). We also prepared solutions of Evans blue at different concentrations and analyzed the fluorescence of these samples by both methods. There was almost no fluorescence detected in samples at concentrations typically delivered to the brain (approximately 20 µg/mL or 0.2% of the injected dose was about 37,000 a.u); however, the fluorescein at the same concentration was 25 times higher (about 970,000 a.u.). This result indicates that either the fluorescence intensity of this dye at such low concentrations is not enough to detect or that the sensitivity of the equipment is insufficient, suggesting that the leakage of Evans blue could have been missed previously due to inadequate detection methods.

### 2.3. Accumulation of Antibody Conjugates in the Brains of Cuprizone-Treated Mice

**Figure 5:**
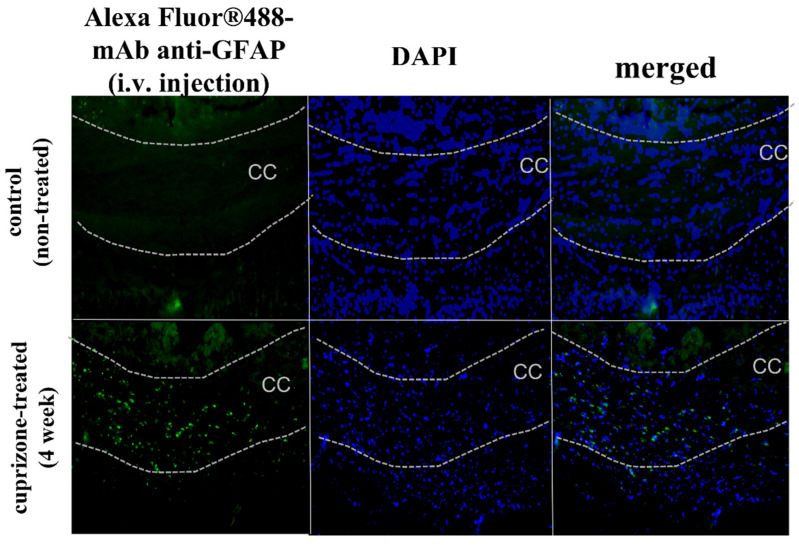
Accumulation of mAb GFAP–Alexa Fluor™ 488 conjugates (green) in the corpus callosum of healthy and cuprizone-treated mice 24 h after intravenous injection as detected by fluorescence microscopy. Nuclei are counterstained with DAPI (blue). Scale bar is 100 µm.

To analyze the accumulation of macromolecular conjugates within the brains of cuprizone-treated mice, we used monoclonal antibodies to GFAP, which were overexpressed in the reactive astrocytes during the 4th week of the diet (Figure S4B). The choice of specific antibodies to GFAP proved crucial, as non-specific IgG exhibited minimal or no accumulation even in a glioma model with compromised BBB function [19,20,21]. So, we used non-specific IgG -conjugate as a control to avoid false-negative results. Employing carbodiimide chemistry, we labeled both the specific mAb GFAP and the non-specific IgG with a fluorescent dye, Alexa Fluor™ 488. At 12 and 24 h after i.v. injection of these fluorescently labeled antibodies, significant accumulation of the specific mAb GFAP was observed in the corpus callosum of cuprizone-treated mice compared to control mice (intact brain), Figure 5. We also observed some penetration through the BBB of non-specific IgG-conjugated contrast agents during the demyelination stage, reflecting increased BBB permeability (Figure S6). Its minor tissue retention could also be explained by the non-specific binding of IgG to the corpus callosum at the demyelination stage, as shown in immunofluorescent analysis (Supplementary Material, Figure S2).

**Figure 6:**
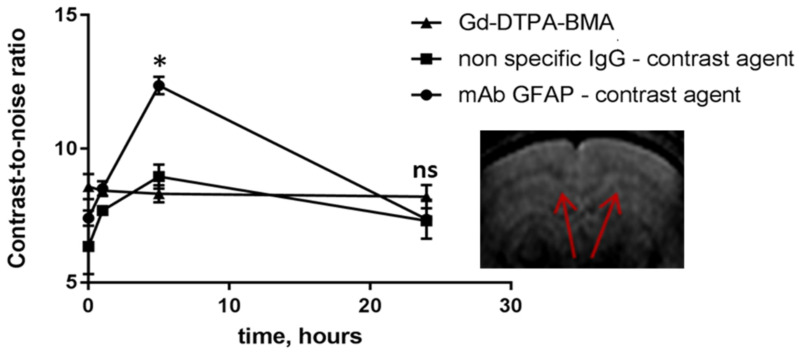
MRI analysis of the CNR values of the corpus callosum after intravenous injection of mAb GFAP-conjugated contrast agent, non-specific IgG-conjugated contrast agent, and Gd–DTPA–BMA (Omniscan^®^) in cuprizone-treated mice after 4 weeks of the diet. Areas of analysis are marked with arrows. Data are presented as mean ± SEM. * *p*-value < 0.05, ns—non-significant.

Then, we synthesized mAb GFAP- and IgG-conjugated gadolinium(Gd)-based contrast agents for MRI monitoring of their accumulation in the brain. We compared the contrast-to-noise ratio (CNR) values in the demyelinated area of the corpus callosum after i.v. injection of the contrast agents. Prior to in vivo administration, we confirmed specificity of mAb GFAP conjugates using immunofluorescent analysis with primary astrocytes (Figure S5). Enhanced accumulation of the specific mAb GFAP-conjugated contrast agent was also observed in the corpus callosum compared to the non-specific IgG contrast agent or the conventional agent Omniscan^®^. The CNR values for both non-specific contrast agents (IgG conjugate and Omniscan^®^) increased to 20% relative to initial CNR values. In contrast, the CNR values for the mAb GFAP-conjugated contrast agent were notably threefold higher than that for controls (non-specific IgG conjugate and Gd–DTPA–BMA) at 5 h post-injection (Figure 6). 

## 3. Discussion

The cuprizone-induced model of demyelination in mice is still among the most popular approaches for the modeling of CNS diseases associated with demyelination and remyelination. However, there is still no consensus on BBB integrity during the cuprizone diet. In fact, there are currently three different opinions on BBB integrity in the cuprizone model: positive («disrupted BBB»), neutral («relatively intact BBB», «limited BBB permeability»), and negative («intact BBB»). This diversity of viewpoints is evident within the literature, and some publications suggest that the cuprizone diet may induce increased BBB permeability [16,17]. Here, we decided to explore the permeability of the BBB in cuprizone-treated mice for macromolecules, particularly antibody conjugates. 

First, we validated our cuprizone-induced model of demyelination by tracking changes using MRI, histological evaluation of mice brains, and accessing gene expression of MBP and PLP proteins in the brain. Previously, we used a 0.2% cuprizone diet for 6 weeks that did not result in any pathological signs of demyelination (no MRI changes, no difference in GFAP-positive cells). We extended the duration of the diet, but the changes were insignificant (still no MRI changes, but a slight increase in GFAP-positive cells). Subsequently, we elevated the cuprizone diet dosage to 0.6% and observed a demyelination pattern comparable to those published previously. This difference in cuprizone dosage and its impact on demyelination might arise due to multiple factors, including strain, gender, source of cuprizone, and supplements in the provided chow. All these elements can impact the severity of the model and should be considered in further investigations. 

The development of pathological lesions in the corpus callosum of cuprizone-fed mice was confirmed by T2-weighted MRI and diffusion tensor imaging after 4 weeks of the cuprizone diet. This process was accompanied by myelin loss (Luxol fast blue staining) and a decrease in mRNA levels of myelin proteins (MBP and PLP gene expression) as detected by histological and rt-PCR analysis. Moreover, we observed a high level of GFAP expression in the corpus callosum using immunohistochemical analysis. It has been shown that cuprizone-induced demyelination accompanies reactive astrogliosis, and an increase in GFAP expression in the grey and white matter of the cerebrum can be detected even after 3 weeks of the 0.2% cuprizone diet in C57BL/6 mice [4]. We observed an enhanced concentration of GFAP-positive astrocytes in demyelinated lesions during the 4th week of demyelination and during the remyelination stage: 2 weeks after diet termination. These results are consistent with the published data confirming that astrocyte reactivity does not normalize rapidly and persists in combination with extensive spontaneous remyelination [27]. 

To study BBB integrity, we analyzed the accumulation of three different macromolecules in the brains of mice treated with cuprizone in comparison with healthy mice. It is known that accumulation of macromolecules is possible only in cases where the BBB has been compromised (e.g., brain injury, brain tumor) [32,33]. Thus, we studied the accumulation of traditional Evans blue, fluorescently labeled antibodies, and antibody-conjugated Gd-based contrast agents using a variety of methods (confocal microscopy, in vivo imaging IVIS, and MRI analysis). 

Evans blue is often used to verify increased permeability of the BBB to macromolecules due to its a very high affinity to serum albumin. Historically, the accumulation of albumin-bound Evans blue is evaluated by extraction of the dye from the brain followed by fluorometric analysis [30]. Four hours after intraperitoneal injection of Evans blue, we did not detect significant differences between the control and cuprizone-treated mice using the standard protocol [34]. Comparable findings were reported by others [15], signifying either limited or weak permeability of albumin-bound Evans blue across the BBB. However, Berghoff et al. demonstrated a minor localized accumulation of Evans blue in a cuprizone-treated brain, implying a slight disruption of BBB integrity [17]. Shelestak et al. (2020) observed an accumulation of Evans blue in an early phase of the cuprizone model [16]. However, it is important to consider the nuances of using of Evans blue as a tracer to assess altered BBB integrity across various studies, for example, variation of injected doses (typically 2% EB, 2–4 mL/kg) and detection methods. In particular, Saunders et al. (2015) criticized the use of Evans blue as a tracer for the assessment of BBB hyperpermeability [35]. They mentioned that high doses of Evans blue lead to its presence as free dye in plasma and its binding to the tissue, which causes misinterpretation of results. As an alternative, they suggested using other tracers with better sensitivity. In alignment with this perspective, we propose that alternative methods are likely to offer enhanced accuracy and the potential to detect even trace amounts of the dye that might have been previously unnoticed. We tested different concentrations of Evans blue to ascertain the minimal detectable concentration for the standard extraction method. We verified that the sensitivity of the standard method (techniques and/or dye) is insufficient for detecting accumulated Evans blue in demyelinated lesions following the cuprizone diet compared to brain tumors, namely glioblastoma multiforme.

In order to capture the subtle accumulation of macromolecules in the brains of cuprizone-treated mice, we intravenously injected monoclonal antibodies to GFAP labeled with the stable and sensitive dye Alexa Fluor 488 and performed confocal analysis of the brain cross sections. In our prior research, we observed minimal accumulation in the brain when employing non-specific antibodies, even in BBB-compromised models like glioma [19,20,21]. To ensure accurate results here, we administered both targeted and non-targeted antibody conjugates within the cuprizone model in this study. Twelve and twenty-four hours after i.v. injection, we observed localized accumulation of the fluorescent-labeled antibodies in the corpus callosum of cuprizone-treated mice compared to control. This localized accumulation may suggest the presence of minor, localized gaps in the BBB within demyelinating areas after micro-necrosis of oligodendrocytes. In addition, the high level of GFAP expression detected in the demyelinated lesions can be associated with increased extracellular space and loss of contacts between astrocytes and oligodendrocytes or astrocytes and myelin sheaths [36]. Recent studies suggested that the mechanism of BBB disruption in the cuprizone model is also associated with microglial and astrocytic activation [37]. Activated astrocytes release cytokines, chemokines, and other factors that contribute to cuprizone-induced demyelination. Petra Fallier-Becker et al. (2022) showed that BBB impairment in the cuprizone model related to changes in astrocyte endfeet and AQP4 isoform expression [38]. Shelestak et al. observed that activation of mast cells was associated with the highest levels of BBB permeability and could potentially mediate BBB disruption [16]. Moreover, we also observed an increased amount of VEGFR2 in the brain during the 4th week of the cuprizone model, which could be associated with BBB disruption, since VEGFR2 is the main signal transducer in endothelial cells [39] and its level indicates the vascular permeability in different models [40], such as brain injury and brain tumors [41]. It was shown that a high amount of VEGFR2 could cause BBB disruption via c-Src activation. 

To confirm fluorescence microscopy results, we performed MRI analysis and compared the CNR and fractional anisotropy values in the brains of cuprizone-fed and healthy mice. In order to trace a macromolecular contrast agent, we conjugated specific monoclonal antibodies to GFAP or non-specific IgG with a Gd-based contrast agent (PLL–DTPA–Gd). MRI analysis demonstrated the accumulation of macromolecular contrast agents in the corpus callosum at the acute phase of demyelination 5 h after i.v. injection, regardless of the specificity of the contrast agent (both specific mAb GFAP and non-specific IgG had increased CNR values). However, the specific GFAP-conjugated contrast agent had enhanced accumulation compared to that of non-specific IgG-conjugated contrast agent, confirming the overexpression of GFAP in the demyelinated lesions. These results could indicate not only local increased BBB permeability in the demyelinated corpus callosum but also the significance of employing targeted tracers for assessing BBB integrity. Moreover, low-molecular-weight contrast agents (Gd–DTPA–BMA) also had slightly increased CNR values at the pathological site. In contrast, Boretius et al. (2012) reported no accumulation of the Gd–DTPA contrast agent in the cuprizone model [41]. However, these variations could potentially arise from subtle changes in CNR that might be challenging to discern visually. 

Our data revealed a local BBB disruption and increased permeability of the BBB to macromolecules, particularly for different antibody conjugates, in the model with elevated cuprizone doses. This was substantiated through fluorescence microscopy and MRI analysis. Intriguingly, we did not observe any detectable amount of Evans blue in the CNS of cuprizone-treated mice using the standard protocol of dye extraction, consistent with existing works in the literature. This could potentially imply that Evans blue extravasation into the brain is limited and subtle, evading detection through conventional protocols. Utilizing technologies and tracers with better detection limits could possibly uncover this marginal BBB permeability more effectively. Thus, employing alternative methods, we were able to detect the accumulation of diverse macromolecules in the brain, suggesting that BBB integrity is compromised in the cuprizone model of demyelination.

## 4. Materials and Methods

### 4.1. Materials

Bis(cyclohexanone)oxaldihydrazone (C9012), gadolinium chloride hexahydrate (GdCl3, G7532), Luxol fast blue solution, non-specific IgG from mouse serum, polylysine (15–30 kD, SIP7890), and diethylenpentaacetic acid (D1133) were purchased from Sigma-Aldrich (St. Louis, MO, USA). Paraformaldehyde was provided by Pancreac (141451.1211). Polyclonal antibodies to glial fibrillary acidic protein (pAb GFAP), monoclonal antibodies to glial fibrillar acidic protein (mAb GFAP), and monoclonal antibodies to vascular endothelial growth factor receptor 2 (mAb VEGFR2) were obtained by hybridoma technology (custom-made materials will be shared upon reasonable request) according to previously published protocol [42,43]. Briefly, mice were immunized by recombinant proteins, isolated splenic B cells were fused with Sp2/0-Ag14 myeloma cells, then hybrid cells were injected and screening of hybridoma cells for selectivity of produced antibodies was performed. Alexa 594™ goat anti-mouse antibodies and Alexa 488™ goat anti-mouse antibodies were obtained from Life Technologies (Carlsbad, CA, USA). Antibodies were validated by their binding efficacy using an ELISA assay.

### 4.2. Experimental Design and Modeling of Cuprizone-Induced Demyelination

All studies on animals were approved by the Ethical Committee of the Serbsky National Medical Research Center for Psychiatry and Narcology (Approval #5). Male C57BL/6 mice were obtained from «Andreevka», Federal Scientific Center of biomedical technologies, Moscow, Russia. All mice were divided into the cage per group (n = 5–7 per group; total number of mice = 60). Demyelination was induced by feeding 8–10 week-old mice with a diet containing 0.6% cuprizone mixed into a ground standard rodent chow for 4 weeks. Control animals were fed powdered chow only. Water was given ad libitum. All animals were weighed every 3 days. To minimize animal suffering during experiments, mice were anesthetized with either isoflurane (MRI study) or zoletil/xylazine anesthesia (other types of experiments). For histological evaluation (Section 4.4, Section 4.5, Section 4.7 and Section 4.8) the mice were anesthetized (Zoletil 50 mg/kg, xylazine 5 mg/kg) and perfused intracardially with 4% paraformaldehyde (PFA) in phosphate-buffered saline (PBS), pH 7.4, at room temperature (RT). Then, brains were removed and kept in 4% PFA in PBS overnight and afterwards in 30% sucrose in PBS for a minimum of 24 h. Coronal sections (20–30 µm) were cut with a cryostat (SLEE medical GmbH, Mainz GmbH, Germany). No exclusion of animals was used in statistical analysis.

### 4.3. rt-PCR Analysis

For gene expression analysis, mice were killed by rapid decapitation. MBP (myelin basic protein), PLP (proteolipid protein), and GFAP (glial fibrillary acidic protein) expression in the brain tissue was assessed after 1, 2, and 4 weeks of the cuprizone diet (demyelination period) as well as 2 weeks after cuprizone withdrawal (remyelination period) by rt-PCR (n = 5 per group). Samples were prepared from brain hemispheres by homogenization in a Tissue Lyser LT (Qiagen, Hilden, Germany). Subsequently, RNA was isolated using the phenol–chloroform extraction method in an automated Qiacube system (Qiagen, USA). RNA concentration was determined sprectrophotometrically by a NanoDrop spectrophotometer (Thermo Fisher, Wilmington, NC, USA). We used 500 ng of total RNA and 20 μL of random decamer primer for the first-strand cDNA synthesis from an RNA template (MMLV RT kit, Evrogen, Moscow, Russia). mRNA levels were normalized to the housekeeping gene (HPRT1) and to the average value of the control group where needed. Specific primers are listed in Supplementary Table S1. Real-time PCR was run on a StepOne instrument (Applied Biosystems, Foster City, CA, USA). The ∆∆Ct method was used to calculate relative expressions of genes of interest (MBP, PLP).

### 4.4. Luxol Fast Blue Staining

Luxol fast blue staining of cuprizone-injured brains was performed to prove demyelination. For this purpose, brain sections were incubated with Luxol fast blue working solution for at least 2–4 h (56 °C) and then standard protocol was followed [44]. Images of the corpus callosum were obtained by light microscopy (Leica, Germany). Quantification of LFB staining was performed using ImageJ 1.52A software (NIH, Bethesda, MD, USA) and calculated as LFB intensity normalized to area.

### 4.5. Immunohistochemistry of GFAP and VEGFR2

Immunofluorescence analysis of GFAP and VEGFR2 was carried out on brain sections of mice with cuprizone-induced demyelination at different time points (1 and 4 weeks of demyelination, 2 weeks of remyelination). For this purpose, standard protocol was followed [43]. Briefly, frozen brain sections with 30 µm thickness were pre-incubated with 5% goat serum (30 min, 37 °C), washed with phosphate-buffered saline (PBS), containing 0.2% Tween 20 and 0.2% Triton X-100 (PBSTT), and incubated with appropriate primary antibodies (pAb GFAP or mAb VEGFR2, 4 °C, overnight). Then, brain sections were washed with PBSTT, incubated with Alexa Fluor 594™ goat anti-rabbit-antibodies (for pAb GFAP) or with Alexa Fluor 594™ goat anti-mouse-antibodies (for mAb VEGFR2), and counterstained with DAPI. Images were obtained using confocal microscopy (Nikon A1 MP, Otawara, Japan). Quantification of GFAP+ cells was determined by applying binary layers to a threshold of the fluorescence intensity. The lower threshold intensity was equated to background noise of control. Next, the area with target cells in µm was analyzed using NIS Elements AR 5.20.02 software.

### 4.6. Magnetic Resonance Imaging (MRI)

To prove demyelination, T2-weighted images were obtained during the 1st, 2nd, and 4th weeks of cuprizone exposure and 2 weeks after diet termination. Animals were anesthetized throughout the whole procedure with the E-Z Anesthesia system (EZ-7000 330, Philadelphia, PA, USA) with 2–3% isoflurane. For T2-weighted images, the Turbo Spin Echo sequence with following parameters was used: TR = 3250 ms, TE = 43, Turbo factor = 9, FOV = 20 × 16.25 mm, base resolution = 192 × 163, number of acquisitions = 5. For T1-weighted images, the FLASH 2D sequence with the following parameters was used: TR = 450 ms, TE = 4.54, flip angle = 70, FOV = 20 × 16.25 mm, slice thickness = 0.7, base resolution = 192 × 163, number of acquisitions = 5. DTI examination was performed with a 7T MR system (ClinScan Bruker BioSpin) with an EPI-SE pulse sequence (TR/TE = 12,000/43 ms; b-factors = 0, 1000, 1500 s/mm^2^; diffusion directions = 12 (Multi-Directional Diffusion Weighting); averages = 2; spectral fat saturation; FOV = 25 × 15.5 mm; slice thickness = 1.0 mm; matrix size = 110 × 68; acquisition time = 10:12). Apparent Diffusion Coefficient (ADC) and Fractional Anisotropy (FA) maps were calculated from DTI data using NUMARIS syngo MR VB15 (Siemens) software.

Efficacy of signal contrast enhancement from pathological lesions was investigated during the 4th week of the cuprizone treatment. For this purpose, GFAP-targeted contrast agents were intravenously (i.v.) injected at a dose of 0.2 mmol Gd/kg. The contrast agents conjugated with non-specific mouse immunoglobulins (IgG) as well as commercial contrast agent OmniScan^®^ (Gd–DTPA–BMA) were used as controls. T1-weighted images were obtained before and 1, 5, and 24 h after injection of contrast agents.

Signal intensities of the injured corpus callosum and other brain tissues (caudoputamen or cortex) of cuprizone-injured mice were measured using SyngoFastViewer (Siemens, Erlangen, Germany) and MultiVox Viewer (GammaMed, Moscow, Russia) software. Signal contrast enhancement from pathological lesions (contrast-to-noise ratio, CNR) for each time point was calculated according to the following equation: CNR = (SI_CC_ − SI noise)/SI noise; SI_CC_—averaged signal intensities of the corpus callosum, SInoise—averaged signal intensities of noise (in the air).

### 4.7. Analysis of Evans Blue Accumulation

Evans blue (2 mg/mL, 20 mL/kg) was intraperitoneally injected (i.p.) to the mice with cuprizone-induced demyelination during the 4th week of modeling. After 3 h, mice were sacrificed and perfused with ice-cold PBS, and brains were monitored using IVIS Spectrum CT. For spectral unmixing of Evans blue fluorescence from tissue auto-fluorescence, the following excitation and emission filters were used: For 605 nm, 660–740 nm emission filters were used. For 640 nm excitation filter, 680–760 nm filters were used. Spectral unmixing was performed using Living Image 4.4 software in manual mode. After IVIS imaging, brains were homogenized, centrifuged, and Evans blue was extracted using trichloroacetic acid as described previously [28].

### 4.8. Accumulation of Antibody Conjugates

To evaluate permeability of the BBB, we injected the following two types of antibody–drug conjugates via the femoral vein: (1) fluorescently labeled mAb GFAP–Alexa 488™ and (2) mAb GFAP labeled with GdCl_3_ by PLL–DTPA linkers. For the first type of conjugate, the standard protocol of conjugation tracers with monoclonal antibodies was followed (Life Technologies, Carlsbad, CA, USA) [45]. Synthesis of the GFAP-targeted contrast agents was conducted as previously described [19]. Briefly, PLL–DTPA conjugates were prepared using a [DTPA]:[Lys] ratio = 1:1. After that, monoclonal antibodies to GFAP (mAb GFAP) were covalently bound with PLL–DTPA (molar ratio [mAb]:[PLL–DTPA] was 1:10) and complexed with GdCl3. The obtained mAb GFAP conjugates were purified by gel filtration chromatography (Sepharose CL-6B, HEPES) from unbounded reagents, sterilized (0.45 µm, Millipore Filter Corp., Bedford, MA, USA), and stored until further use. Selective binding of conjugated antibodies to GFAP was studied using immunofluorescence analysis on primary astrocytes.

### 4.9. Statistical Analysis

Statistical analysis was performed using GraphPrism 8 software. One-way ANOVA with Bonferroni correction were used to analyze the data sets. No randomization was performed to allocate subjects in the study. No exclusion criteria were pre-determined.

## Data Availability

The datasets used and/or analyzed during the current study are available from the corresponding author on reasonable request.

## Supplementary Materials

The following supporting information can be downloaded at: https://www.mdpi.com/article/10.3390/ijms241612688/s1.
