# Supplementary material for: Localized Increased Permeability of Blood–Brain Barrier for Antibody Conjugates in the Cuprizone Model of Demyelination
Source: Int J Mol Sci. 2023 Aug 11;24(16):12688. doi: 10.3390/ijms241612688 (PMC10454543; doi:10.3390/ijms241612688)
Supplement: Supplementary file 1 [file ijms-24-12688-s001.zip › ijms-2500202-supplementary.pdf]

Supplementary materials

Localized Increased Permeability of Blood--Brain Barrier for Antibody Conjugates in the Cuprizone Model of Demyelination

Tatiana Abakumova, Anastasia Kuzkina, Philipp Koshkin, Daria Pozdeeva, Maxim Abakumov, Pavel Melnikov, Klavdia Ionova, Ilia Gubskii, Olga Gurina, Natalia Nukolova and Vladimir Chekhonin

Table S1. Specific primers for qPCR used in the study

	**Forward primer**	**Reverse primer**
MBP	GACTCACACACGAGAACTACC	GAAGAAATGGACTACTGGGTTT
PLP	ACCTGTTTATTGCTGCGTTTGTG	TTAAGGACGGCGAAGTTGTAAGT
HPRT	CGAGGAGTCCTGTTGATGTTG	GATAAGCGACAATCTACCAGAGG

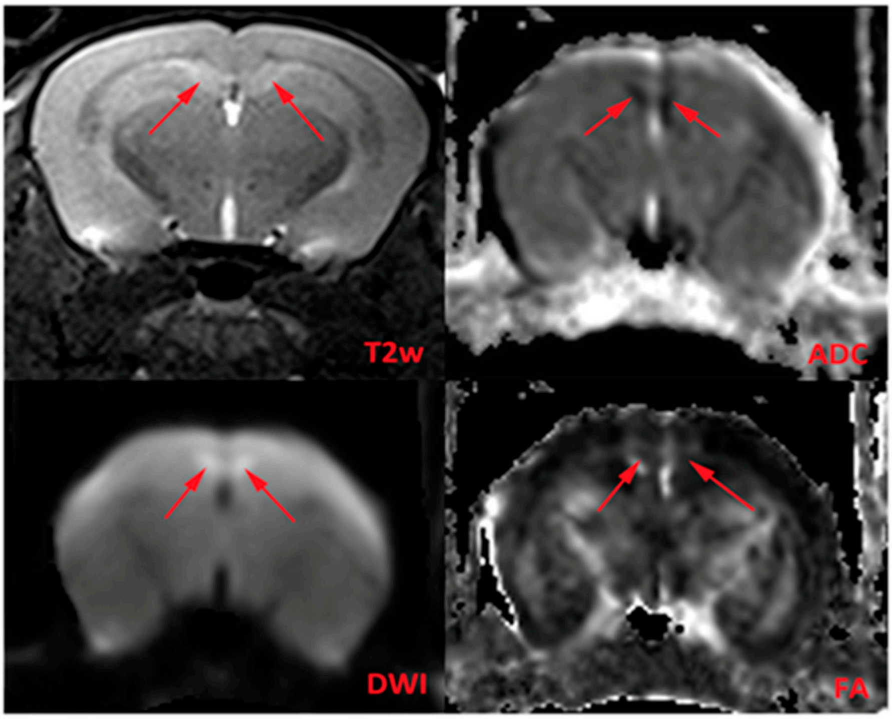

Figure S1. MRI images of cuprizone-treated mice at 4 week of diet: T2w- conventional T2-imaging, FA – fractional anisotropy, DWI- diffusion tensor imaging, ADC – Apparent Diffusion Coefficient. Pathological areas are marked with red arrows.

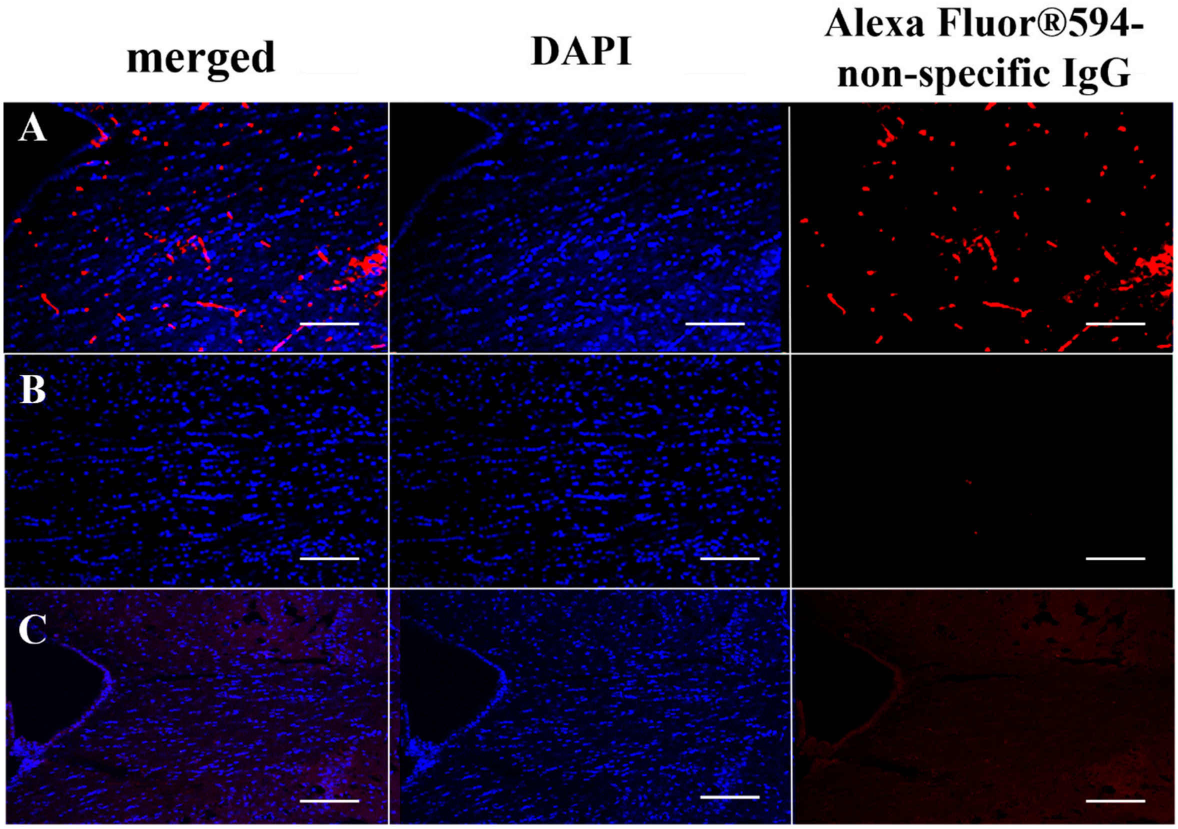

Figure S2. Non-specific IgG were used as control for demyelinated (A) and remyelinated (B) brain in immunohistochemical analysis of cuprizone-treated mice and non-treated healthy control mice (C)

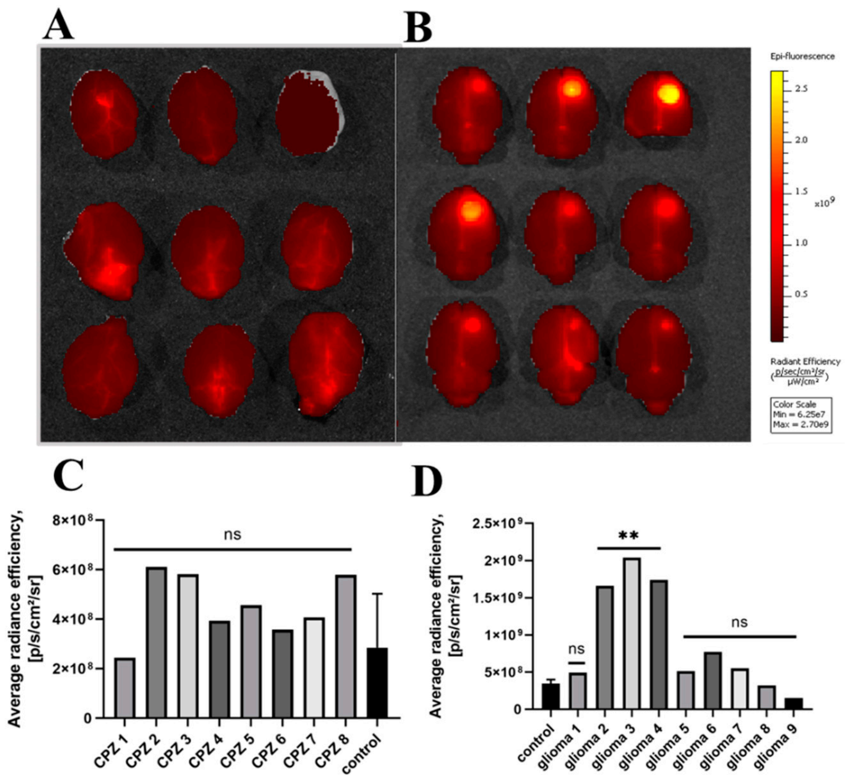

Figure S3. Evans blue accumulation in cuprizone-intoxicated mice (A,C) and C6 glioma (B,D) model using IVIS Spectrum CT. **-p-value < 0.005, ns-non-significant (in comparison with control)

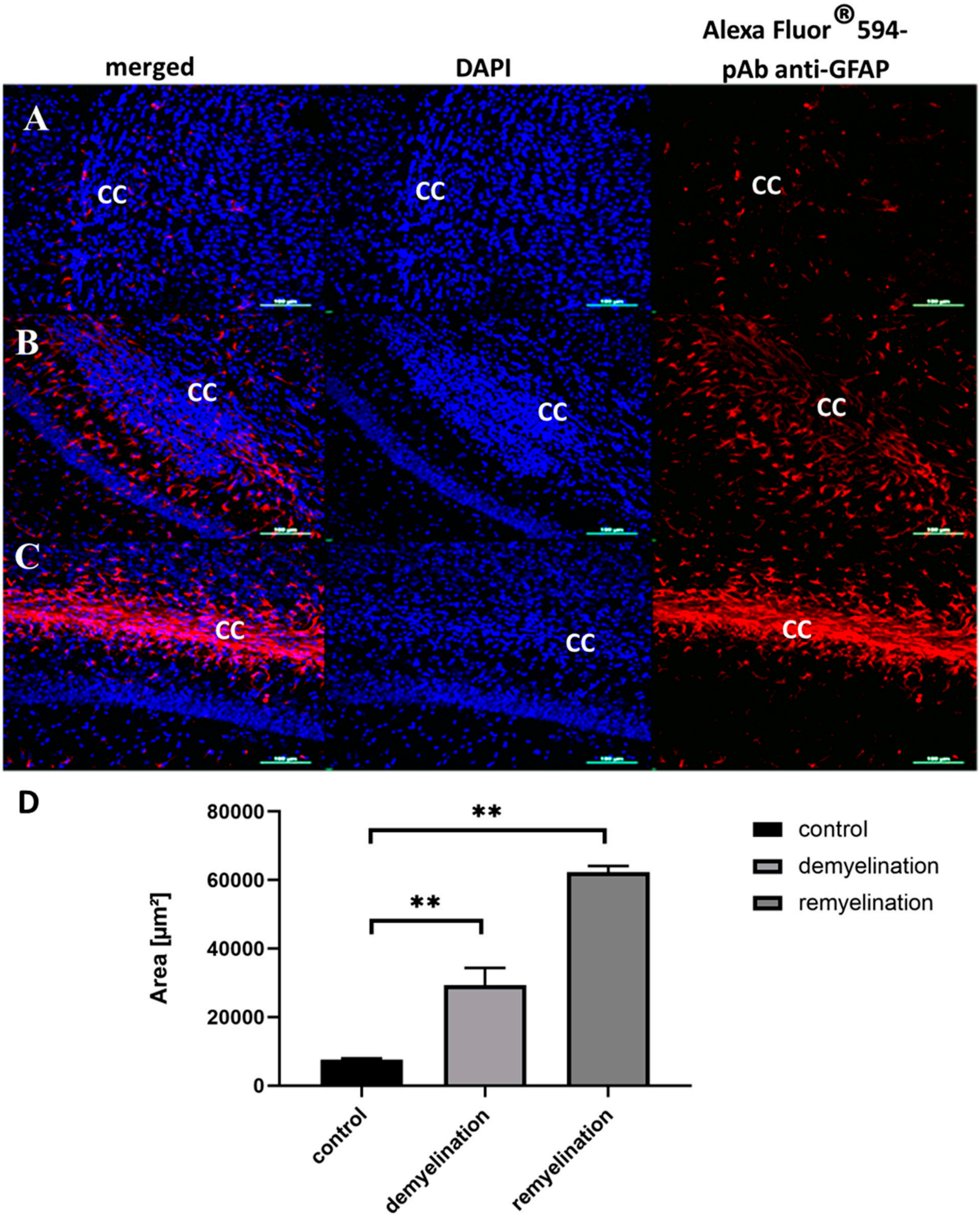

Figure S4. Immunofluorescence staining with pAb anti-GFAP of the corpus callosum (CC) of the healthy (A) and cuprizone-treated mice on the 4th week of demyelination (B) and at the remyelination stage (in 2 weeks after cuprizone withdrawal) (C). Scale bar is 500 µm. Quantification analysis of area of GFAP+ cells at immunofluorescent tissue staining of control (healthy) and cuprizone-treated mice at demyelination (4 week) and remyelination stage (D). ** p-value < 0.01

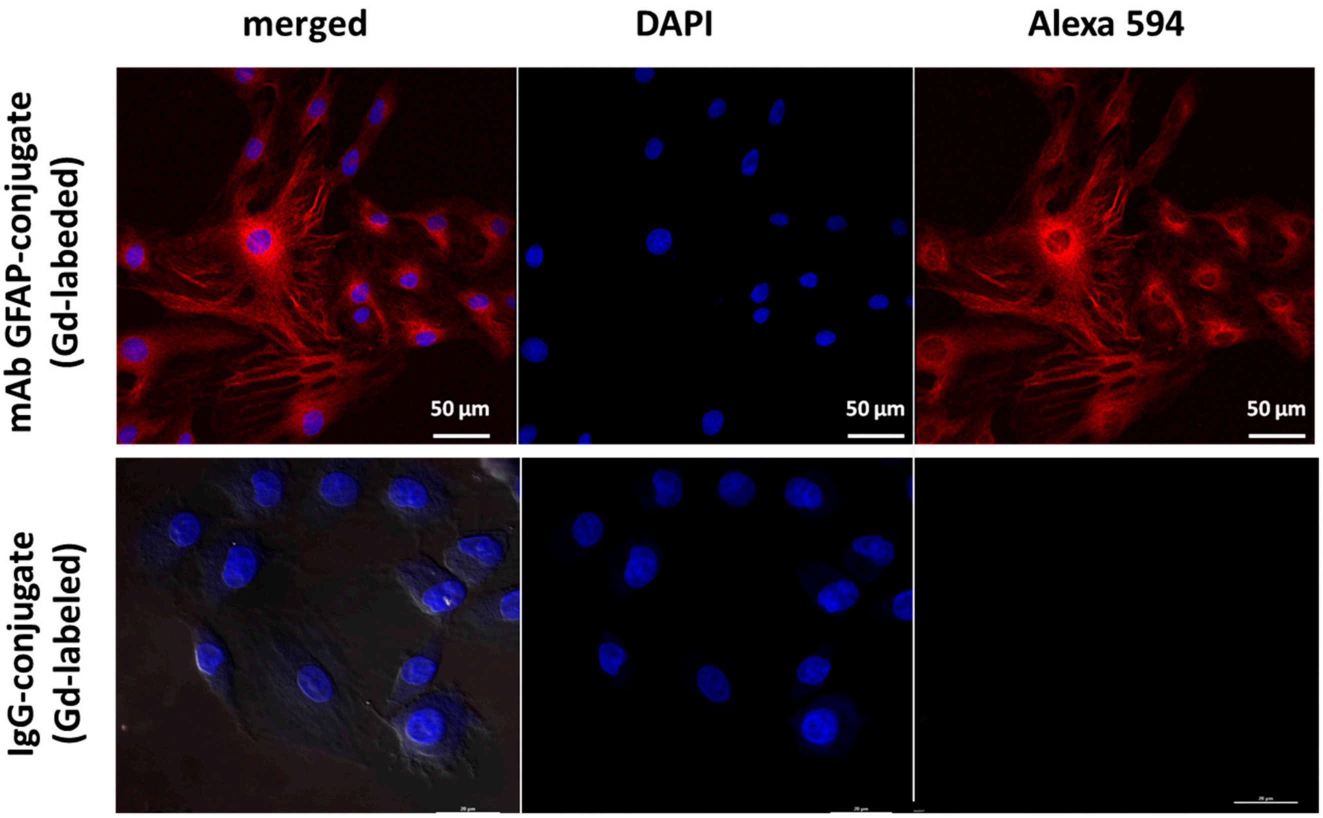

Figure S5. Immunofluorescent analysis of Gd-labeled monoclonal antibodies to GFAP and Gd-labeled non-specific IgG on primary astrocytes.

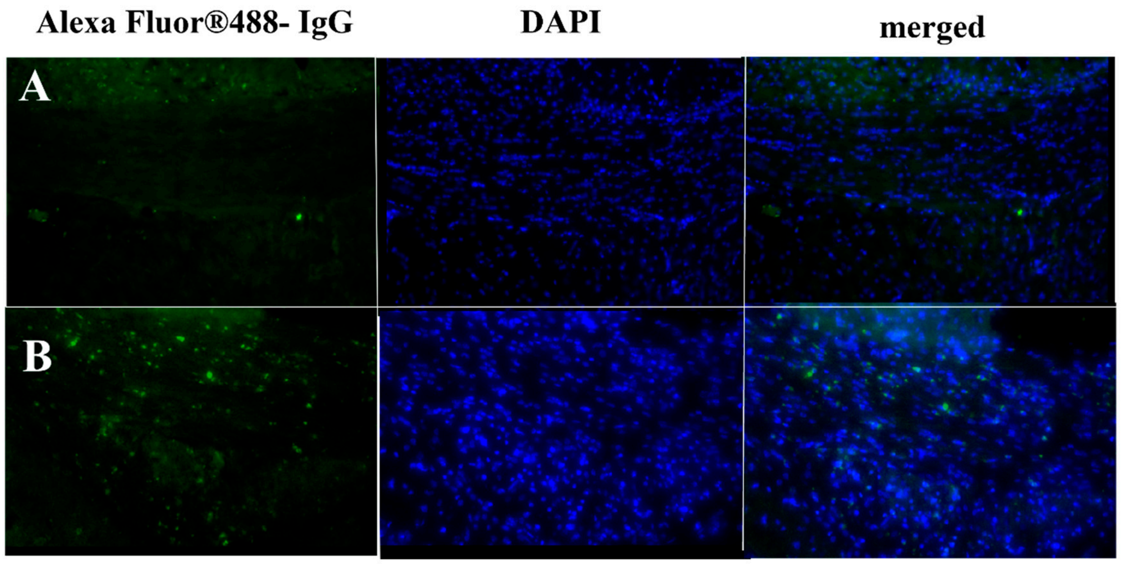

Figure S6. Accumulation of non-specific IgG -Alexa Fluor™ 488 conjugates in the corpus callosum of healthy (A) and cuprizone-treated mice (B).

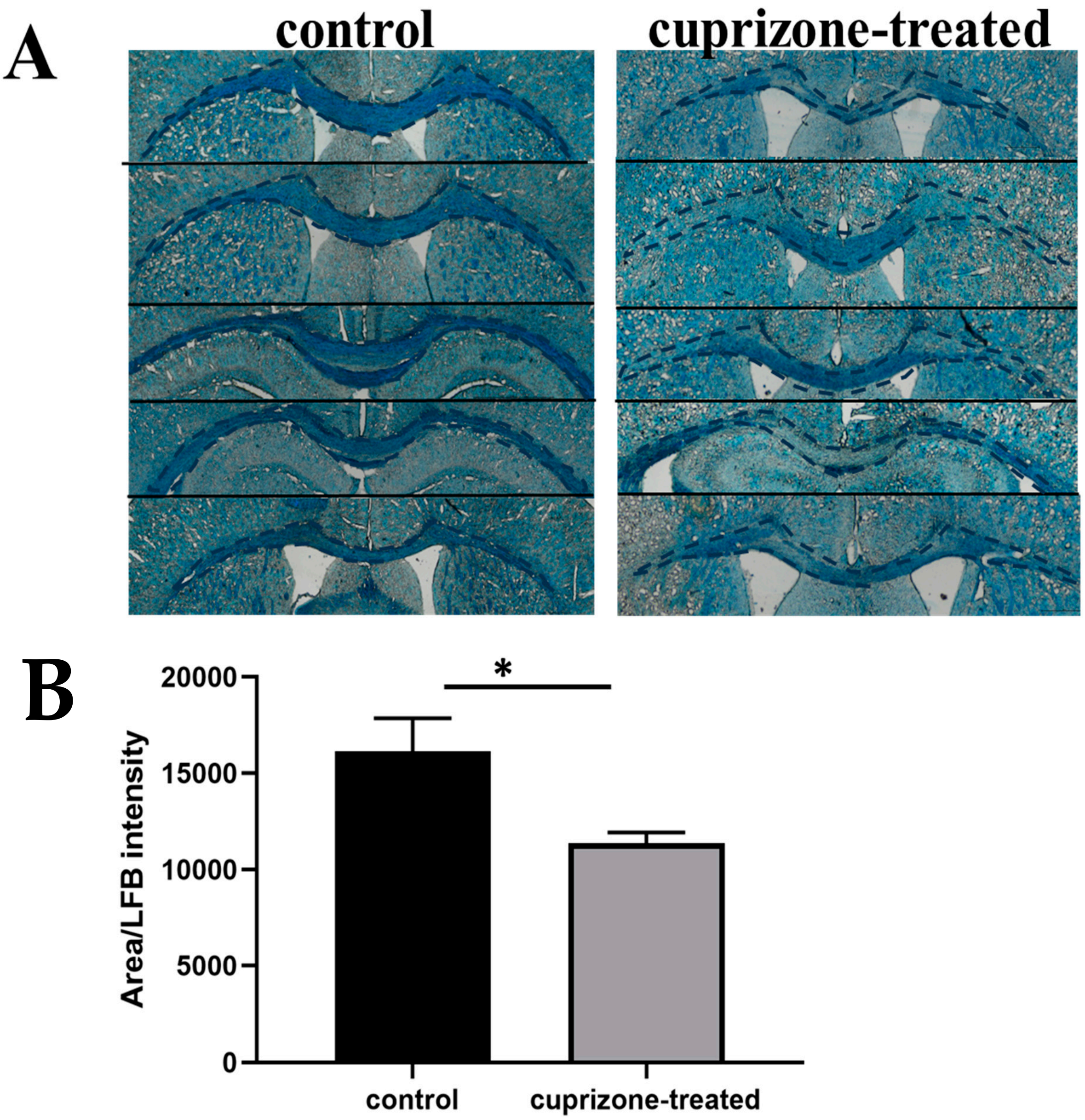

Figure S7. Luxol Fast blue staining of control and cuprizone-treated mice at different levels (A) and quantification of LFB staining (B).* p-value < 0.05.
